# Attitudes of home-visiting nurses toward risk management of patient safety incidents in Japan

**DOI:** 10.1186/s12912-022-00905-2

**Published:** 2022-06-06

**Authors:** Keiko Yoshimatsu, Hisae Nakatani

**Affiliations:** 1 Department of Nursing, Faculty of Nursing and Nutrition, The University of Shimane, 151 Nisihayasigi-cho, Izumo-shi, Shimane-ken 693-8550 Japan; 2 Department of Community and Public Health Nursing, Graduate School of Biomedical and Health Sciences, Hiroshima University, 1-2-3 Kasumi, Minami-ku, Hiroshima City, 734-8551 Japan

**Keywords:** Home-visiting nurses, Risk management, Patient safety incidents, Incident prevention, Home care, Attitudes, Qualitative descriptive approach

## Abstract

**Background:**

In situations of home care, patients and their family members must address problems and emergencies themselves. For this reason, home-visiting nurses (HVNs) must practice risk management to ensure that patients can continue receiving care in the comfort of their homes. The purpose of this study was to examine HVNs’ attitudes toward risk management.

**Methods:**

This study adopted a qualitative description approach. Semi-structured interviews were conducted to collect information on HVNs’ risk management behavior and their attitudes toward it. Participants comprised 11 HVNs working at home-visiting nursing agencies in a prefecture of Japan. Transcribed interviews were analyzed using content analysis.

**Results:**

Nurses’ attitudes toward risk management comprised the following themes: (i) predicting and avoiding risks, (ii) ensuring medical safety in home settings, (iii) coping with incidents, and (iv) playing the role of administrators in medical safety, which was answered only by administrators.

**Conclusions:**

When practicing risk management, home-visiting nurses should first assess the level of understanding of the patient and family, followed by developing safety measures tailored to their everyday needs. These results further suggest that administrators should take actions to foster a working environment conducive to risk management. These actions include coordinating duties to mitigate risk and improve the process of reporting risks. This study provides a baseline for future researchers to assist patients and families requiring medical care services of this nature.

## Background

Japan has a growing population of older adults (aged 65 years or above), leading to increasing numbers of people requiring long-term nursing care [1]. In response to this issue, the government has outlined the “Community-based Integrated Care System,” through which older adults can receive long-term care while continuing to live in their own homes and neighborhoods [2]. However, home care entails a problem: because private homes have no permanently stationed medical staff as hospitals do, the older person and their family must cope with problems by themselves. For this reason, home-visiting nurses (HVNs) must practice risk management to ensure that the person can continue receiving care at home, with peace of mind.

Patient safety aims to prevent risks, errors, and harms pertaining to patients, and the basis of the safety measures is to learn from errors and adverse events [3]. Based on the previous literature [4–7], risk management was defined as an individual or organizational effort to prevent incidents. Patient safety was defined as preventing risks, errors, and harms associated with patients and their families. Incidents are events that cause or potentially may cause unnecessary harm to a patient. Half the patients who receive home care after being discharged experience adverse events and require interventions to avoid them [8]. In Japan, the occurrence of adverse events in home-care nursing agencies is low. However, there is no standardized reporting system [9], and it is possible that all events have not been reported. For patient safety in home care services, it is important to have regular error management training [10]. Home-visiting nursing agencies in Japan provide training in the prevention of medical incidents and produce incident reports. In a study on home care workers in Sweden, Larsson et al. [11] found that these workers struggle to perform risk assessments and follow safety regulations due to a “lack of time, equipment and information” (p. 342). In Japan, these agencies are typically small, with an average of only 4.8 HVNs [12]. Therefore, we speculate that it may be difficult to conduct a risk assessment at each patient’s home visit because of the high number of visits that must be completed. Nevertheless, HVNs must be able to identify and address potential dangers in the home care setting in order to prevent medical incidents. However, agencies struggle to carry out systematic preventive procedures because, unlike hospitals, HVNs provide care at the patient’s home.

In the home-visiting nursing agency, the HVNs, under the supervision of the administrator, consider the patient’s health and safety in a way that reflects the features of the home-visiting nursing service. However, administrators have heavy workloads and inadequate support, and consequently, experience more stress than staff nurses in ensuring medical safety [13]. Despins [14] noted the important role that hospital staff nurses play in the early detection and mitigation of patient risks and revealed organizational and individual attributes that impacted this role. Similarly, in home-visiting nursing, the systematic and individual factors of home-visiting nursing are thought to affect the early detection and reduction of patient risk. Thus, the importance of risk management needs to be recognized by both administrators and HVNs, who, together, must practice risk management to ensure the patient’s safety. Studies have reported that there is a need to improve patient safety in home care settings [9, 15]. However, although adverse events in the home environment are likely to occur due to human factors, it has been pointed out that there is a lack of research on aspects such as risk management and human factors related to nurses [16]. In home care, care providers are not limited to medical professionals but include family caregivers too. HVNs spend less time at the patient’s home. Therefore, patients spend overwhelmingly more time with family members who are not medical professionals. There is a high possibility that an incident such as a fall will occur while the HVN is absent. However, incidents that occurred when the HVN was absent are often not recorded, and the challenge is to build a system that can accumulate and analyze information for taking preventive measures [17]. Therefore, adverse events and incidents in Japan may not be accurately understood. In the absence of a standardized reporting system, it may be up to the individual nurse’s thoughts and sensitivities to report the incidents that the HVN has experienced and discovered. Therefore, it is important to understand HVNs’ perspective on patient safety in a home care environment. Nurses with a positive attitude towards safety have less experience with adverse events. Therefore, nurses need to improve their attitude towards safety in order to promote a positive safety culture [18]. Along these lines, promoting a positive safety culture requires understanding HVNs’ attitudes towards patient safety, awareness, feelings, and behaviors related to risk managment. Individual factors such as attitude involve cognitive, emotional, and behavioral components [19]. Attitudes, mainly, are often considered based on cognitive and emotional components. However, they influence behavioral intent and should be considered based on three factors [20]. As an educational evaluation of patient safety, the participant’s attitude toward patient safety may be used [21, 22]. For patient safety in home care, it is important to clarify how the HVN recognizes patient safety and leads to behavior. In this study, the HVN’s attitude toward patient safety was defined as the HVN’s perceptions, feelings, and behaviors related to patient safety. Therefore, to help home-visiting nursing agencies develop effective safety procedures, it is necessary to identify the attitudes that HVNs exhibit in their efforts to prevent medical incidents. Thus, this study aimed to identify HVNs’ attitudes toward risk management.

## Methods

### Design

This exploratory study adopted a qualitative approach using semi-structured interviews. HVNs’ risk management attitudes can clarify how HVNs perceive patient safety and can shape HVN risk management behaviors. This naturalistic approach deepens understandings of such little-known human phenomena through the analysis and interpretation of the meanings participants attribute to events [23]. Therefore, we adopted this approach to clarify HVN’s risk management behaviors and the perceptions and emotions that lead to those behaviors. The study period was from May 2016 to May 2017. Data were collected from HVNs in one prefecture in western Japan. This prefecture has coastal areas, mountains, and remote islands, and was selected because it allowed us to reflect the characteristics of various regions.

The certification criteria stipulate that each home-visiting nursing agency must have at least 2.5 full-time equivalent (FTE) nurses. Each administrator should be a nurse and be in charge of the agency’s management and activities, recruit service users, ensure efficient mobilization of staff, and coordinate with other community resources [24]. In Japan, most administrators provide home-visit nursing services, similar to nurses, owing to labor shortages.

### Participants

**Fig. 1:**
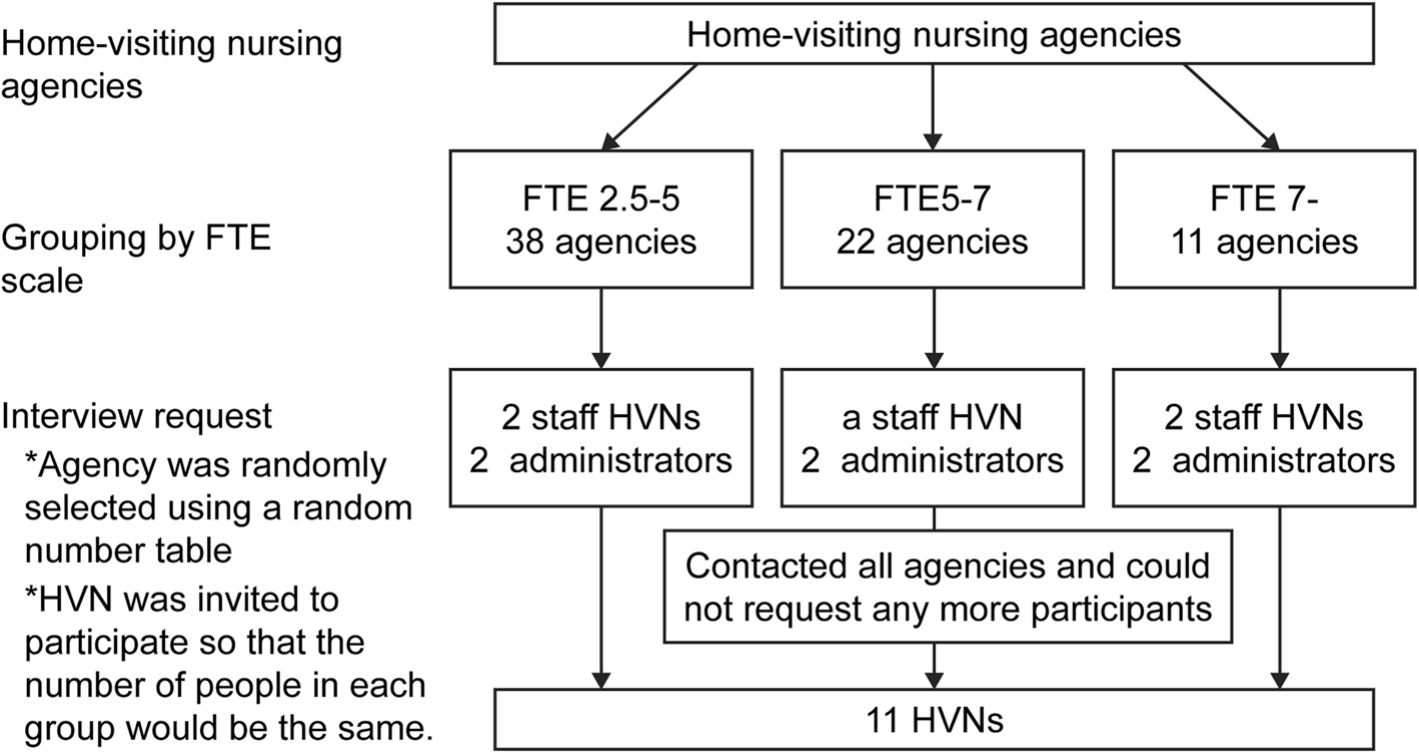
Flow chart of participant recruitment and participation

HVNs from 71 home-visiting nursing agencies in a prefecture of Japan were randomly selected using a random number table (Fig. 1). The agencies were divided into three groups by size: those with FTEs of 2.5–5, 5–7, and > 7 nurses. HVNs were invited to participate so that the number of people in each group would be the same. Groups with FTEs of 2.5–5 and 7 and above were represented by two staff HVNs and two home-visiting nursing administrators. In the group with FTEs of 5–7 nurses, as a result of asking all the agencies in the group, an HVN and two home-visiting nursing administrators agreed to participate. Finally, eleven people from six agencies participated in this study. Participants comprised 5 HVNs and 6 home-nursing administrators working at home-visiting nursing agencies in a prefecture of Japan. Since HVNs visit a patient’s home alone, the risk to the patient needs to be assessed on the spot. Nurses who have been recognized as capable of visiting a patient’s home personally are considered to have practice regarding medical safety. Therefore, participants were required to be HVNs with the skills necessary to be able to perform home-visit nursing alone.

### Process

Each participant attended a semi-structured interview lasting 40 to 60 minutes. Interviews were conducted at participants’ workplaces, as per their wish, by one representative researcher (RN, MSN). The representative researcher, who is female, has experience as an HVN and learned about research methods in graduate school. The following information was obtained from the HVN of administrators and staff HVNs: “awareness of risk management for patient safety,” “risk management efforts such as incident prevention” and “thoughts on the risk management efforts that HVN is practicing.” During the interviews, participants freely described their behaviors towards patient safety and how they felt about them. An interview guide was created through a discussion between researchers on the basis of a previous study [13].

### Analysis

With the participants’ permission, the interviews were recorded and transcribed. The transcripts were subjected to content analysis, with a focus on context. The qualitative content analysis process was conducted in accordance with the method presented by Graneheim and Lundman [25]. First, the researcher read through the entire transcript several times to get an overall sense of it. Subsequently, texts on HVNs’ attitudes toward risk management were extracted and divided into condensed semantic units. The narratives were coded into basic units of meaning, each consisting of a word and phrase that was understandable by itself. Interview data were individually analyzed after collection. After coding all the transcripts, the code list was created. After the analysis of 11 interviews, the researchers concluded that no new category would emerge even if we continued the interview. The saturation of data was thus confirmed. Next, units that were similar or related were grouped together into subcategories. Finally, the subcategories were compared in terms of their similarities, relatedness, and differences, and further grouped into categories. Coding was performed by one representative researcher, followed by another researcher who reviewed it. In addition, the two researchers collaborated to negotiate and improve the findings to ensure the validity of the analytical process. The research results were shared with the research team as part of a discussion and quality check until the final theme was decided. All these steps were recorded. The analyzed content was shared with the participants. It was confirmed that all the participants agreed to the contents of the analysis. This study was reported according to the COREQ guidelines [26].

### Ethics

Ethical approval was obtained from the Independent Ethics Committee of The University of Shimane, Japan (study approval number: 191). All methods were performed in accordance with the Declaration of Helsinki. The participants received a verbal and written briefing that outlined the study’s purpose. The briefing also conveyed to the participants that their participation was entirely voluntary, they could withdraw their consent at any time, their anonymity would be maintained, and that the study may be published in journals or presented at conferences. Informed consent was confirmed in writing.

## Results

### Participant characteristics

Interview data were obtained from 11 HVNs. Table 1 shows the basic attributes of the participating HVNs, who had an average age of 51.7 ± 6.4 years. Six of the participants were administrators and five were staff HVNs. All administrators provided home-visit nursing services to the homes of patients. All participants were working full-time. The agencies to which participating HVNs with FTEs of 2.5–5 and 5–7 belonged were half in urban or coastal areas and half in mountainous areas. In contrast, if the FTE scale of the agency to which the participating HVN belonged was 7 or above, the agencies were in urban areas.

**Table 1:** Home-visiting nurses’ basic data

**Attribute**	**A**	**B**	**C**	**D**	**E**	**F**
Gender	F	F	F	F	F	F
Employment position	administrators	administrators	administrators	administrators	administrators	administrators
Age	50s	50s	50s	50s	50s	40s
Years in nursing	22	35	25	33.5	25.5	27
Years as HVN	3.5	18	20	2.5	8 months	22
Type of employment	Full-time	Full-time	Full-time	Full-time	Full-time	Full-time
FTE nurses in agency	6.8	8.2	8.7	4	3.4	6
**Attribute**	**G**	**H**	**I**	**J**	**K**	
Gender	F	F	F	F	F	
Employment position	staff HVN	staff HVN	staff HVN	staff HVN	staff HVN	
Age	40s	30s	50s	50s	50s	
Years in nursing	24	10	21	26	18	
Years as HVN	15	6 months	20	19	12	
Type of employment	Full-time	Full-time	Full-time	Full-time	Full-time	
FTE nurses in agency	5.5	4	3.4	8.7	8.2	

*F* Female, *HVN* Home-visiting nurse, *FTE* Full-time equivalent

### Attitudes of HVNs toward risk management

**Fig. 2:**
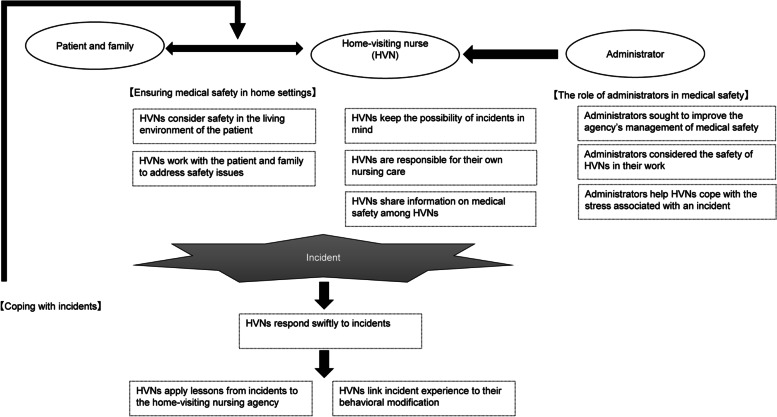
Attitudes of HVNs toward risk management

HVNs’ attitudes toward risk management comprised the following themes: (i) predicting and avoiding risks, (ii) ensuring medical safety in home settings, (iii) coping with incidents, and (iv) playing the role of administrators in medical safety (Table 2). Figure 2 shows the attitudes of HVNs toward risk management. Home visiting nursing was conducted “Predicting and avoiding risks” for medical safety. In addition, by caring for the patient, they were “Ensuring medical safety in home settings.” When a harmful incident occurred, HVNs responded promptly to the incident and conducted “Coping with incidents” to improve operations. Further, administrators performed HVN activities by “Playing the role of administrators in medical safety.”

**Table 2:** Attitudes of HVNs toward risk management

Category	Sub-category
Predicting and avoiding risks	HVNs keep the possibility of incidents in mind
	HVNs ensure that they take responsibility for their own nursing care
	HVNs share information on medical safety among HVNs
Ensuring medical safety in home settings	HVNs consider safety in the living environment of the patient
	HVNs work with the patient and family to address safety issues
Coping with incidents	HVNs respond swiftly to incidents
	HVNs apply lessons from incidents to the home-visiting nursing agency
	HVNs link incident experience to their behavioral modification
The role of administrators in medical safety	Administrators sought to improve the agency’s management of medical safety
	Administrators considered the safety of HVNs in their work
	Administrators help HVNs cope with the stress associated with an incident

HVNs: home-visiting nurses

### Predicting and avoiding risks

This category comprised three subcategories.

#### HVNs keep the possibility of incidents in mind

The HVNs were aware of the possibility of being involved in an incident and maintained a constant awareness of medical safety.

*“I always think that the care I provide may lead to incidents, and I try to prevent a harmful incident.” (Staff HVN K).*

*“Since I am a nurse, I always think about the possible dangers/risks related to the place I am going to visit, and if I am worried or not confident about it, I try to address the issue before going there.” (Administrator A).*

#### HVNs ensure that they take responsibility for their own nursing care

The HVNs only visited the home once they were confident about how to use the medical equipment, and they allowed themselves enough time to handle the situation with care. To avoid a dangerous complacency that might arise from growing accustomed to routine behaviors, the HVNs focused on their care activities to maintain a sense of responsibility. They also reflected on what they learned in medical training seminars and applied it to their practice.

*“I’m afraid that my behavior becomes habitual, so I try to focus on what I’m doing.” (Staff HVN J).*

The administrators’ top priority was preventing medical incidents and mastering knowledge and skills regarding the latest care practices in order to avoid incidents. They believed that because HVNs typically work alone, they should take responsibility for their nursing care by taking sufficient time and conducting their duties calmly.

*“I believe that risk management is the most important thing to do as an HVN. Therefore, I try to always learn techniques and gain knowledge to avoid harmful incidents.” (Administrator A).*

#### HVNs share information on medical safety among HVNs

Some of the staff at the home-visiting nursing agencies had difficulty attending training workshops because they worked part-time, with reduced working hours, to raise children. The administrators emphasized the importance of all HVNs being aware of risks and never overlooking them. To that end, they took steps to ensure that the content of medical safety workshops was communicated to all HVNs; for example, those who attended the workshops would hold seminars or produce written reports to communicate the contents.

*“The person in charge of medical safety participates in the training. Following which, they conduct a study session in the office—where, they teach me what was taught during the training.” (Staff HVN K).*

*“I plan a study session so that when the staff nurse receives training, I can inform all staff. For part-time staff who have difficulty participating in training [because of] childrearing, I am preparing to read the report and share the contents.” (Administrator C).*

### Ensuring medical safety in home settings

This category comprised two subcategories.

#### HVNs consider safety in the living environment of the patient

Since HVNs provide care in a home setting, the risks may not be obvious. The patient can decide whether to accept the care recommended by the HVN. Therefore, HVNs considered safety issues by giving due consideration to the patient’s feelings and way of life. To that end, HVNs pointed out the difficulty of recognizing the problems and dangers associated with home care and noted the importance of identifying and providing care in a way that reflects the level of understanding of the patient and family. The HVNs considered a broad range of incidents, including those involving patient accidents/injuries and those involving damage to property or traffic accidents. Concerning the impact of incidents, they considered the effects that such incidents would have on both the patient and on the home-visiting nursing agency.

*“Since the patient lives at home, there are some areas where the lifestyle cannot be changed. I can’t enforce it, so I try to convey the risks of living to patients.” (Staff HVN J).*

*“I understand that HVNs consider risks and provide care conscientiously. However, it is impossible for HVNs to care for a patient while respecting his or her life and feelings without the support of an administrator.” (Administrator F).*

#### HVNs work with the patient and family to address safety issues

The HVNs emphasized the importance of ensuring that the patient and family avoid repeating the same mistake. They worked with the patient and family on safety measures that they could easily implement. To that end, the HVNs built a rapport with the patient and family.

*“When a patient makes a mistake, I will check [on] how well the patient understands the event, and I will discuss with the patient how to avoid the same mistake.” (Staff HVN I).*

The administrators emphasized the importance of considering safety issues together so that a patient and the family could see the issues as matters that concern them.

*“I work hard to appeal to my patients and families to work together and stay safe.” (Administrator C).*

### Coping with incidents

This category comprised three subcategories.

#### HVNs respond swiftly to incidents

The HVNs’ top priority was responding swiftly to incidents in order to achieve a timely resolution. They regularly worked with other professionals in order to keep the patient out of danger.

*“When an incident occurs, I will set the safety of the patient as the top priority and will contact the administrator first to respond.” (Staff HVN K).*

“*When a problem occurs, I think it is important to respond early and provide peace of mind.”* (Administrator C).

#### HVNs apply lessons from incidents to the home-visiting nursing agency

The HVNs emphasized that an incident involving a single HVN is a matter for the home-visiting nursing agency as a whole. Accordingly, the HVNs regularly updated each other about the situation during home visits, and they would confer with each other rather than making decisions independently. Since the process of identifying an incident was different for each HVN, they shared their own assessment of the situation with one another. The HVNs worked to create an environment in which they could discuss incidents frankly. To mitigate the stress associated with incident reports, they reminded each other that the purpose of reporting is not to impute blame and used a simplified method for writing up the reports.

*“I think it’s good to be able to talk about what to do in the future and how to deal with it, rather than limiting the experience of an individual to just one.” (Staff HVN G).*

Administrators also encouraged healthy working relationships between HVNs and promoted a workplace in which HVNs could report and discuss incidents frankly. Additionally, they emphasized the importance of evaluating incident responses and using insights to develop manuals or corrective actions.

*“For home-visiting nursing agencies, it is important to change manuals and improve work by applying lessons from incidents as lessons learned.” (Administrator F).*

#### HVNs link incident experience to their behavioral modification

The HVNs tried to turn an incident into a positive experience by proactively reporting it and taking action to prevent reoccurrence. By reviewing the factors that caused the incident and the way they handled it, they identified ways to change their behavior.

*“When I experience an incident, I think it’s important to look back, report, and avoid repeating it.” (Staff HVN G).*

The administrators treated incidents seriously and took steps to prevent similar incidents from occurring in the future. The administrators reflected upon themselves in the incident report, which led to behavior modification. Administrators took the initiative in making the incident report easier for HVNs to write.

*“When I experience an incident, I first look back on myself.” (Administrator D).*

### The role of administrators in medical safety

This category comprised three subcategories, all of which were only regarding administrators’ narratives.

#### Administrators sought to improve the agency’s management of medical safety

The administrators believed that HVNs should, amid their hectic schedule, consider medical safety issues and that they should be constantly mindful of incidents so that they would actively collect safety information. They highlighted the possibility that some incidents might go unreported because HVNs each have a different attitude toward incidents. To address this problem, they routinely exhorted HVNs to confer with them about incidents so that they could handle them as administrators. When unsure about how to deal with an incident, the administrators would consult a supervisor and work with other home-visiting nursing agencies.

*“I have a responsibility as a manager of the home-visiting nursing agency, so I encourage staff to consult, contact, and report.” (Administrator D).*

#### Administrators considered the safety of HVNs in their work

Administrators considered how HVNs handled their duties, considered their health conditions, and coordinated their duties to enable them to work safely. They also informed HVNs of what they deemed to be dangers and risks.

*“I try not to make mistakes [with] staff nurses, to be careful about fatigue, and adjust my work so that I can work in good health.” (Administrator A).*

#### Administrators help HVNs cope with the stress associated with an incident

Administrators highlighted a problem related to the small size of home-visiting nursing agencies: if an HVN is identified as the person who caused an incident, he or she might experience severe stress. Accordingly, in order to mitigate the worry and stress associated with incident reports, the administrators endeavored to maintain anonymity in the reporting process and emphasize that the purpose of the report was not to impute blame. They also listened carefully to the HVN, ensuring that the person had enough time to collect their thoughts.

*“Being a small business, it is mentally painful for the nurse to speak about what actually happened... Since improving operations is important, I consider similar cases and keep the parties to the incident, undisclosed.” (administrator F).*

## Discussion

HVNs practiced the prevention of harmful medical incidents, which included predicting potential medical incidents, post-incident response, and evaluation. According to Lang [27], safety-related interventions in home health care do not allow supporters to choose how patients and their families live and must coordinate patient/family-centered care according to the patient’s perspective of vulnerabilities, needs, and strengths. Our results showed that HVNs in Japan face several challenges in home care that differ from those associated with in-hospital care. Therefore, HVN practiced risk management of patient safety to understand and act on these issues to prevent adverse events for patients.

### Predicting and avoiding risks

HVNs emphasized the importance of self-improvement and taking responsibility for one’s nursing practice. According to Schildmeijer et al. [28], adverse events among home care patients are common and mostly preventable, and addressing these requires improved interprofessional collaboration. HVN behavior in our study is consistent with this finding as it was found important to always be aware of incidents and share safety information between HVNs and with other occupations.

According to Tong et al. [29], despite safety concerns, patients and family caregivers prefer to receive home care. In the future, demand for home care will increase further, and it is expected that HVNs will work in various employment forms at home-visiting nursing agencies. According to Andersson et al. [30], home-visit nursing requires the transfer of mutual knowledge between nurses, which leads to quality care and treatment of nurses. The administrators considered the background of nurses, such as working part-time to raise children, so that all staff could share information. It is necessary to establish a system that allows all HVNs to share knowledge and take responsibility for care.

### Ensuring medical safety in home settings

HVNs were aware that since the risks they identified existed in the patient’s home, they could not act heavy-handedly and force the patient to take safety measures. For example, HVNs in Japan must be mindful of the possibility that (i) the patient may refuse to stop prohibited behaviors, (ii) the circumstances behind an incident may be unclear, and (iii) an incident may be caused by the use of the patient’s own equipment or belongings.

Another feature of home-visiting services is the fact that HVNs, in contributing to the patient and family’s quality of life, must respect the patient’s autonomy and lifestyle. Rather than addressing safety in a heavy-handed manner, HVNs must accept the patient’s choices and possibly allow the patient to maintain their behavior, or at least mitigate the risks associated with it. Therefore, as part of risk management, HVNs noted the importance of assessing the level of understanding of patients and their families and developing a safety plan that fits their lifestyle.

In a study on the safety experiences of family caregivers in the context of home mechanical ventilation, Schaepe and Ewers [31] concluded that nurses and other health professionals “should act in partnership with family caregivers and allow them to deliberately choose their role in patient care and safety” (p. 9). In addition, according to Sears et al. [32], improving patient and caregiver education, skill development, and clinical planning could be useful interventions to reduce harmful incidents. According to Sahlström et al. [33], patient-reported safety incidents suggest a practical system-based solution to prevent recurrences. In this study, HVNs emphasized the importance of considering the safety issues together so that a patient and family could see them as matters that concern them. It is essential for HVNs to ascertain the extent to which the patient and family understand the risks and to actively communicate the risks in a way that makes the patient and family appreciate how they are matters of their concern. To that end, HVNs should identify the risks to the patient and family and encourage them to undertake preventive actions in their home life. Therefore, HVNs must comprehend the capacity of the patient and family to understand the issues and circumstances in the home care environment and have the communication skills necessary to share information and support the patient and family.

### Coping with incidents

When the administrator and HVNs experienced an incident, they prioritized ensuring patient safety. In addition, both the administrators and HVNs sought to correct and improve operations at their home-visiting nursing agency. According to Maurits et al. [34], good communication and discussion of incidents among health professionals can help reduce mistakes, but nurses at home health care institutions have difficulty with reporting mistakes to colleagues. Recognition of the incident and the need for an incident report varied between HVNs. Therefore, the administrator acted as a role model by proactively reporting incidents. According to Johannessen et al. [35], administrators and staff need to be more involved in developing strategies to understand work practices, challenges, and risks, and learn from positive deviations and what works for patients during risk management. In this study, both administrators and HVNs considered that it was important to have active communication between HVNs for information exchange in risk management. Therefore, it is important to foster a workplace safety culture in which the experience of incidents can be communicated to staff and used as an educational opportunity to improve future work.

### The role of administrators in medical safety

The administrators, in developing their agency’s procedures for managing medical safety, considered both the patient and family’s safety as well as approaches for ensuring that HVNs can administer their services safely. This finding is consistent with a report by Ree and Wiig [36], which identified teamwork in home healthcare as a “significant contributing factor to patient safety” (p. 1) and argued that “building sound teams with mutual trust and collaboration should therefore be an essential part of managers’ work” (p. 1). Supplementing HVNs’ efforts to identify and treat risks, administrators improved the home-visiting nursing agency’s safety procedures, coordinated HVNs’ duties, and mitigated the stress associated with incidents. Andersson et al. [30] highlighted the importance of such actions by arguing that managers should be aware of nurses’ competencies and provide nurses with sufficient support, as this will help prevent burnout and staff turnover. Indeed, the administrators communicated closely with the HVNs, assessed their awareness of medical safety, and helped them take practical steps to improve safety.

However, a challenge was also highlighted in this process: when it came to reporting incidents, HVNs recognize the need to report adverse events, but these events are underreported because of shame [37]. In this study, HVNs experienced stress related to the lack of anonymity in reporting, given that home-visiting nursing agencies are typically small. This finding suggests that in addition to administrators informing HVNs that incident reports are not intended to impute blame, HVNs should regularly remind each other of this fact and exchange information about patients in order to foster a climate where HVNs can report incidents frankly.

In terms of relevance to clinical practice, our findings suggest that administrators in all home-nursing agencies would benefit from taking action to promote a work environment that is conducive to risk management. For this, it was suggested that it is necessary to improve the working environment, for example, by establishing a process for HVNs to report risks. This study provides a baseline for future researchers to build on for the benefit of patients and families requiring medical care services of this nature.

### Limitations

The present study had some limitations. First, in this study, data were collected from participants in urban areas and mountainous areas (agencies with FTE 2.5–5 and 5–7) and participants in urban areas only (agencies with FTE 7 and above). There was no difference in narratives by region. However, the sample was from a single region (i.e., a prefecture in western Japan). Since the results may reflect regional factors, there may be a limit to how far they can be generalized. Thus, future research should use a sample from a broader geographical area. Second, there is a possibility that HVNs who were interested in risk management participated in the interview, implying selection bias. Finally, in the absence of information to triangulate our findings, it is possible that participants provided information to portray themselves in a good light (i.e., a social desirability bias).

## Conclusion

Administrators and staff provided information regarding how they identified and treated potential medical incidents, how they dealt with incidents that have occurred, and how they evaluated the way they handled the incidents. HVNs were aware that since the risks they identify exist in the patient’s home, they cannot act heavy-handedly and force the patient to take safety measures. Instead, HVNs respected the patient and family’s feelings and way of life and worked with them to find an agreeable safety plan. Thus, as part of their risk management, HVNs must assess the level of understanding of the patient and family and then develop a safety plan that comports with their way of life. In order to prevent adverse incidents, home-visit nursing requires not only professionals but also patients and families to work together as members of the team. Therefore, we encourage HVNs to improve their communication skills to identify risks with patients and their families.

## Data Availability

The datasets supporting the conclusions of this article are included within the article and its additional files.

## Abbreviations

HVN: Home-visiting nurse
FTE: Full-time equivalent
